# Enzyme‐Cleaved Bone Marrow Transplantation Improves the Engraftment of Bone Marrow Mesenchymal Stem Cells

**DOI:** 10.1002/jbm4.10722

**Published:** 2023-02-11

**Authors:** Hotaka Kawai, May Wathone Oo, Kiyofumi Takabatake, Ikue Tosa, Yamin Soe, Htoo Shwe Eain, Sho Sanou, Shigeko Fushimi, Shintaro Sukegawa, Keisuke Nakano, Takarada Takeshi, Hitoshi Nagatsuka

**Affiliations:** ^1^ Department of Oral Pathology and Medicine, Graduate School of Medicine, Dentistry and Pharmaceutical Sciences Okayama University Okayama Japan; ^2^ Department of Regenerative Science, Graduate School of Medicine, Dentistry and Pharmaceutical Sciences Okayama University Okayama Japan; ^3^ Cartilage Biology and Regenerative Medicine Laboratory, College of Dental Medicine Columbia University Irving Medical Center New York NY USA; ^4^ Department of Oral and Maxillofacial Surgery Kagawa Prefectural Central Hospital Takamatsu Japan

**Keywords:** BONE FORMATION, BONE MARROW MESENCHYMAL STEM CELLS, BONE MARROW TRANSPLANTATION MODEL, OSTEOBLASTS, SYSTEM BIOLOGY—BONE INTERACTOR

## Abstract

Mesenchymal stem cell (MSC) therapy is a promising approach to curing bone diseases and disorders. In treating genetic bone disorders, MSC therapy is local or systemic transplantation of isolated and in vitro proliferated MSC rather than bone marrow transplantation. Recent evidence showed that bone marrow MSC engraftment to bone regeneration has been controversial in animal and human studies. Here, our modified bone marrow transplantation (BMT) method solved this problem. Like routine BMT, our modified method involves three steps: (i) isolation of bone marrow cells from the donor, (ii) whole‐body lethal irradiation to the recipient, and (iii) injection of isolated bone marrow cells into irradiated recipient mice via the tail vein. The significant modification is imported at the bone marrow isolation step. While the bone marrow cells are flushed out from the bone marrow with the medium in routine BMT, we applied the enzymes’ (collagenase type 4 and dispase) integrated medium to wash out the bone marrow cells. Then, cells were incubated in enzyme integrated solution at 37°C for 10 minutes. This modification designated BMT as collagenase‐integrated BMT (c‐BMT). Notably, successful engraftment of bone marrow MSC to the new bone formation, such as osteoblasts and chondrocytes, occurs in c‐BMT mice, whereas routine BMT mice do not recruit bone marrow MSC. Indeed, flow cytometry data showed that c‐BMT includes a higher proportion of LepR^+^, CD51^+^, or RUNX2^+^ non‐hematopoietic cells than BMT. These findings suggested that c‐BMT is a time‐efficient and more reliable technique that ensures the disaggregation and collection of bone marrow stem cells and engraftment of bone marrow MSC to the recipient. Hence, we proposed that c‐BMT might be a promising approach to curing genetic bone disorders. © 2023 The Authors. *JBMR Plus* published by Wiley Periodicals LLC on behalf of American Society for Bone and Mineral Research.

## Graphical abstract

Enzyme‐cleaved bone marrow isolation favors the isolation of bone marrow mesenchymal stem cells along with hematopoietic stem cells. Isolation of bone marrow cells using a normal culture medium (upper panel) is difficult to separate the bone marrow cell aggregates containing bone marrow mesenchymal stem cells (MSCs) and hematopoietic stem cells (HSCs). The filtration limits the bone marrow cell aggregates from passing through. Applying enzymes such as collagenase and dispase (lower panel) dissociates the aggregation of MSCs and HSCs into single cells and facilitates the filtration step.

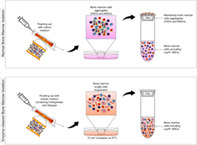

## Introduction

The adult bone marrow is a reservoir of stem cells essential for tissue repair and regeneration.^(^ ^1^, ^2^, ^3^ ^)^ The stem cells can self‐renew and differentiate into multiple mature cell types. Bone marrow stem cells include hematopoietic stem cells (HSCs) and mesenchymal stem cells (MSCs). HSCs are a heterogeneous population of progenitor cells giving rise to mature blood cells in a well‐defined hierarchy. Bone marrow‐derived MSCs (BMSC) have been shown to differentiate into bone, cartilage, and fat.^(^ ^4^, ^5^ ^)^ Therefore, MSCs therapy became extremely widespread in treating bone diseases and disorders.

Bone fracture is the most common medical condition, and its consequences include nonunion and delayed union. Systemic bone disorders such as osteoporosis and Paget's diseases are common aging‐related disorders, resulting in pathological bone fractures and altered bone‐healing processes. These medical conditions are related to deteriorated equilibrium of bone formation and resorption. Recent studies have revealed that systemic and local MSC therapies improve the bone‐healing process, and transplanted MSC showed cartilage regeneration and osteoblast differentiation.^(^ ^6^ ^)^ A genetic disorder of bone known as osteogenesis imperfecta is caused by a mutation in collagen production genes (COL1A1 or COL1A2 gene), resulting in type I collagen defect and bone fragility increase. A cure for genetic bone disorders does not exist yet. However, recent preclinical and clinical studies have revealed that intrauterine or local transplantation of MSC showed successful engraftment in both mouse and human models of osteogenesis imperfecta.^(^ ^7^, ^8^, ^9^, ^10^ ^)^

Systemic MSC transplantation is performed by intravenous or intra‐arterial injection of MSC in humans and animals. In mice, bone marrow transplantation (BMT) via the tail vein is favorably applicable to trace the plasticity of bone marrow‐derived cells in diseases including bone healing and cancers.^(^ ^11^, ^12^, ^13^, ^14^, ^15^ ^)^ The studies on BMT models have shown that bone marrow‐derived cells are distributed to myofibroblasts and fibroblasts in multiple organs, chondrocytes, and osteoblasts in bone, and tumor endothelium and cancer‐associated stromal cells in the tumor microenvironment.^(^ ^16^ ^)^ However, some studies on BMT models revealed that bone marrow cells did not differentiate into osteoblasts. These findings suggested that there may have been an unsolving problem in the BMT technique.

In this study, we present the novel modified technique of BMT to solve the controversial findings of bone marrow‐derived cell involvement in bone regeneration. To identify the bone marrow (BM) cells' involvement in bone regeneration, we created ectopic bone formation models on BMT mice. We compared the modified BMT to routine BMT in which BM cells from donor mice were collected by flushing out with HBSS solution and transplanted to recipient mice via the tail vein. In our modified BMT method, we applied the solution containing collagenase and dispase to collect the BM cells from donor mice. Compared with routine BMT techniques, our modified BMT technique showed higher recruitment of bone marrow‐derived stromal cells and differentiation of BM‐derived cells to osteoblasts and cartilaginous cells, whereas the routine BMT technique failed to recruit the osteoblast precursor from bone marrow.

## Materials and Methods

### Mice

Female mice (GFP transgenic mice, C57BL/6‐Tg [CAG‐EGFP] OsbC14‐Y01‐FM131, and C57BL/6 wild‐type mice) were purchased from Shimizu Laboratory Suppliers (Kyoto, Japan) and were housed under pathogen‐free conditions. All animal experiments were undertaken by the guidelines of the Okayama University Care and Use of Laboratory Animals. This research was approved by the Committee on the Ethics of Animal Experiments of Okayama University Graduate School of Medicine, Dentistry and Pharmaceutical Sciences (OKU ‐ 2020 096).

### Bone marrow transplantation (BMT)

BMT was carried out as described previously.^(^ ^16^ ^)^ The BMT method is divided into three parts: (i) isolation of BM cells from donor mice, (ii) whole‐body lethal irradiation to the recipient mice, and (iii) injection of isolated BM cells into irradiated recipient mice. At first, BM cells were freshly collected from 8‐week‐old GFP transgenic mice. We collected the femur and tibia from mice and removed the soft tissues from the bone. Then, proximal parts of bone tissues were cut. The BM space was washed out with HBSS (Gibco, Thermo Fisher Scientific, Waltham, MA, USA) until the bone color turned white. The collected BM cells were filtered with a cell strainer (100 μm, BD Falcon, BD, Franklin Lakes, NJ, USA) and resuspended within HBSS (Gibco) at a concentration of approximately 1.0 × 10^7^ cells/0.2 mL. Subsequently, 8‐week‐old female C57BL/6 recipient mice underwent 10 Gy of lethal whole‐body irradiation, and collected BM cells were injected into the recipient mice via the tail vein.

### Collagenase bone marrow transplantation (c‐BMT)

In this experiment, we modified the BMT method, especially at the step of BM cells isolation. Unlike the routine BMT described above, we collected the BM cells by flushing out the BM with a solution of 10 mL of HBSS (Gibco) containing collagenase type 4 (10 mg, Gibco) and dispase (20 mg, Gibco) and were incubated at 37°C for 10 minutes. Then, cells were filtered and resuspended within HBSS (Gibco) at a concentration of approximately 1.0 × 10^7^ cells/0.2 mL. Then, BM cells were injected and transplanted into the irradiated recipient mice via the tail vein. We named the modified bone marrow transplantation method collagenase‐treated BMT (c‐BMT), while the routine method is BMT.

### Implantation procedure

The implantation procedure was performed after 1 month of BMT or c‐BMT. In this experiment, to induce ectopic bone formation, recombinant human bone morphogenetic protein‐2 (rhBMP‐2) and insoluble bone matrix (IBM) were used as described previously.^(^ ^11^ ^)^ An amount of 150 mg IBM loaded with 10 μg of rhBMP‐2 (PeproTech, RockyHill, NJ, USA) was implanted into the mouse femoral muscle. Mice were euthanized after 1 month for histological observation.

### Tissue processing for histological examination

For the preparation of formalin‐fixed paraffin‐embedded sections, the harvested tumor and bone were fixed in 4% paraformaldehyde for 12 hours. Decalcification of bone was performed in 10% EDTA at 4°C for 14 days. Then, samples were consequentially dehydrated in 70% ethanol and embedded in paraffin. Serial sections (7 μm) were prepared. Sections were stained with H&E, IHC, and fluorescent IHC.

### Immunohistochemistry (IHC)

IHC was carried out using the antibodies detailed in Supplemental Table S1. The sections were deparaffinized in a series of xylene for 15 minutes and rehydrated in graded ethanol solutions. Endogenous peroxidase activity was blocked by incubating the sections in 0.3% H_2_O_2_ in methanol for 30 minutes. After the antigen retrieval, sections were treated with 10% normal serum for 15 minutes and then incubated with primary antibodies at 4°C overnight. Signals were enhanced by the avidin‐biotin complex method (Vector Lab, Burlingame, CA, USA). Color development was performed with DAB (Histofine DAB substrate), and sections were counterstained with Myer's hematoxylin. The staining results were observed with an optical microscope (BX53, Olympus, Tokyo, Japan).

### Double‐fluorescent IHC

Double‐fluorescent IHC for GFP/RUNX2, GFP/Osteocalcin (OC), and GFP/Sox9 was performed using the primary antibody to GFP, RUNX2, osteocalcin (OC), and Sox9 listed in Supplemental Table S1. The secondary antibodies used are listed in Supplemental Table S2. After the antigen retrieval, the sections were incubated in Block Ace (DS Pharma Biomedical, Osaka, Japan) for 20 minutes at room temperature (RT). The sections were incubated with secondary antibodies for 1 hour at RT. After the reaction, the sections were stained with DAPI. The staining results were observed with an All‐in‐One BZ x700 fluorescence microscope (Keyence, Osaka, Japan).

### Flow cytometry

After isolation of BM cells from donor mice, flow cytometric analysis was performed to compare the BM cell populations of the BMT and c‐BMT methods. After lysing RBCs with ammonium chloride, cells were filtered through a cell strainer (100 μm, BD Falcon). Cells (1.0 × 10^7^) were suspended in 100 μL of 2% FBS/PBS and then incubated with anti‐leptin receptor (LepR) biotinylated antibody (R&D Systems, Minneapolis, MN, USA; BAF497, 1:100), anti‐CD51 antibody (BioLegend, San Diego, CA, USA; 104105, 1:100), anti‐CD45 antibody (BioLegend, 103111, 1:100), anti‐TER‐119 antibody (BioLegend, 116211, 1:100), and anti‐CD31 antibody (BioLegend, 102509, 1:100) for 60 minutes on ice. Then cells were washed and incubated with streptavidin‐BV421 antibody (BioLegend, 405226, 1:1000) for 20 minutes on ice. For RUNX2 detection, cells were fixed and permeabilized with a transcription factor buffer set (BD Biosciences, San Jose, CA, USA) and incubated with an anti‐RUNX2 antibody (Cell Signaling, Danvers, MA, USA; #12556 S, 1:200) for 30 minutes on ice. Then cells were washed and incubated with anti‐rabbit antibody (Cell Signaling, #4412 S, 1:400) for 30 minutes on ice. Fluorescence was detected using a MACSQuant (Miltenyi Biotec, Bergisch Gladbach, Germany). Data analysis was performed using FlowJo software (BD Biosciences).

### Quantification and statistical analysis

At the ectopic bone formation site, we performed quantification and found the average of the five captured images of the healing area at the magnification of 400× per mouse (*n* = 5). The counting and area measurement was performed using ImageJ (v1.52a). And statistical analyses were conducted using GraphPad Prism 9.1.1 (GraphPad, La Jolla, CA, USA). In short, a two‐tailed Student's *t* test for independent samples with equal variances was used to compare two groups. Differences were considered significant at *p* < 0.05. Data were presented as mean ± SD.

## Results

### c‐BMT shows the well‐established bone marrow transplantation in mice

**Fig. 1:**
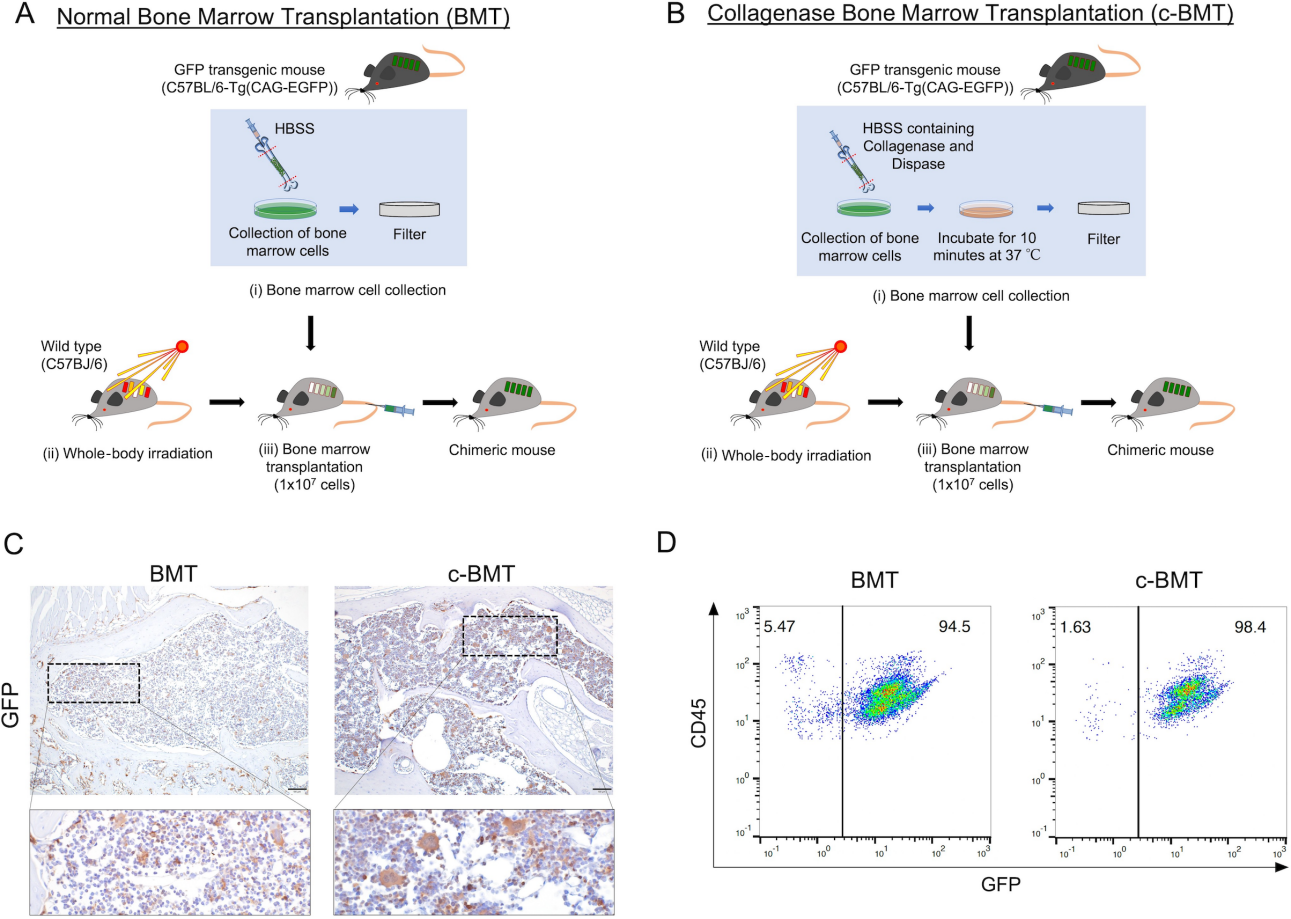
Illustration of bone marrow transplantation (BMT). (*A*) Illustration of normal BMT (BMT) method. (*B*) Illustration of modified BMT (c‐BMT) method. (*C*) Representative immunohistochemical (IHC) images of GFP detection on recipient mice femur showing the total engraftment of donor bone marrow cells. Left panel: BMT; right panel: c‐BMT. Scale bar = 100 μm. (*D*) Scatter plots from flow cytometry analysis of isolated bone marrow cells depicting CD45‐positive and GFP^+^.

As illustrated in Fig. 1, we modified the routine BMT method (Fig. 1 *A*, *B*). Briefly, we modified the isolation of bone marrow cells from the donor. In bone marrow transplantation, we first isolated the bone marrow cells from the donor while the recipient mice were irradiated. Then, isolated bone marrow cells were transplanted to the recipient mice via the tail vein. In normal BMT method, we isolated the bone marrow cells by flushing out the bone marrow with HBSS (Fig. 1*A*). In our modification, we flushed out and incubated the bone marrow with the medium incorporated with collagenase and dispase (Fig. 1*B*). Here, we defined the optimal concentration of collagenase and dispase for the medium preparation to wash out the cells from the BM. As shown in Supplemental Fig. S1, we tested different combinations of collagenase and dispase and evaluated the proportion of BMSC in the isolated bone marrow cells. In comparing the addition of different graded collagenase and dispase, adding 10 mg of collagenase and 20 mg of dispase to 10 mL of HBSS showed the optimal collection of LepR^+^ BMSC. In the adult bone marrow, the LepR^+^ BMSC population is the major source of osteoblasts, chondrocytes, and adipocytes.^(^ ^17^ ^)^ After BMT, we confirmed the establishment of BMT and c‐BMT in mice by GFP IHC staining on the femur and flow cytometry analysis. All BMT and c‐BMT mice demonstrated the complete replacement of BM with GFP^+^ cells (Fig. 1*C*). In addition, flow cytometry data showed 94.5% and 98.4% of CD45^+^ bone marrow cells were GFP^+^ in BMT and c‐BMT mice, respectively (Fig. 1*D*). These data suggested that our modified c‐BMT method is reliable and well established as a routine BMT method and useful for future BMT models.

### Ectopic bone formation occurred in both BMT and c‐BMT

**Fig. 2:**
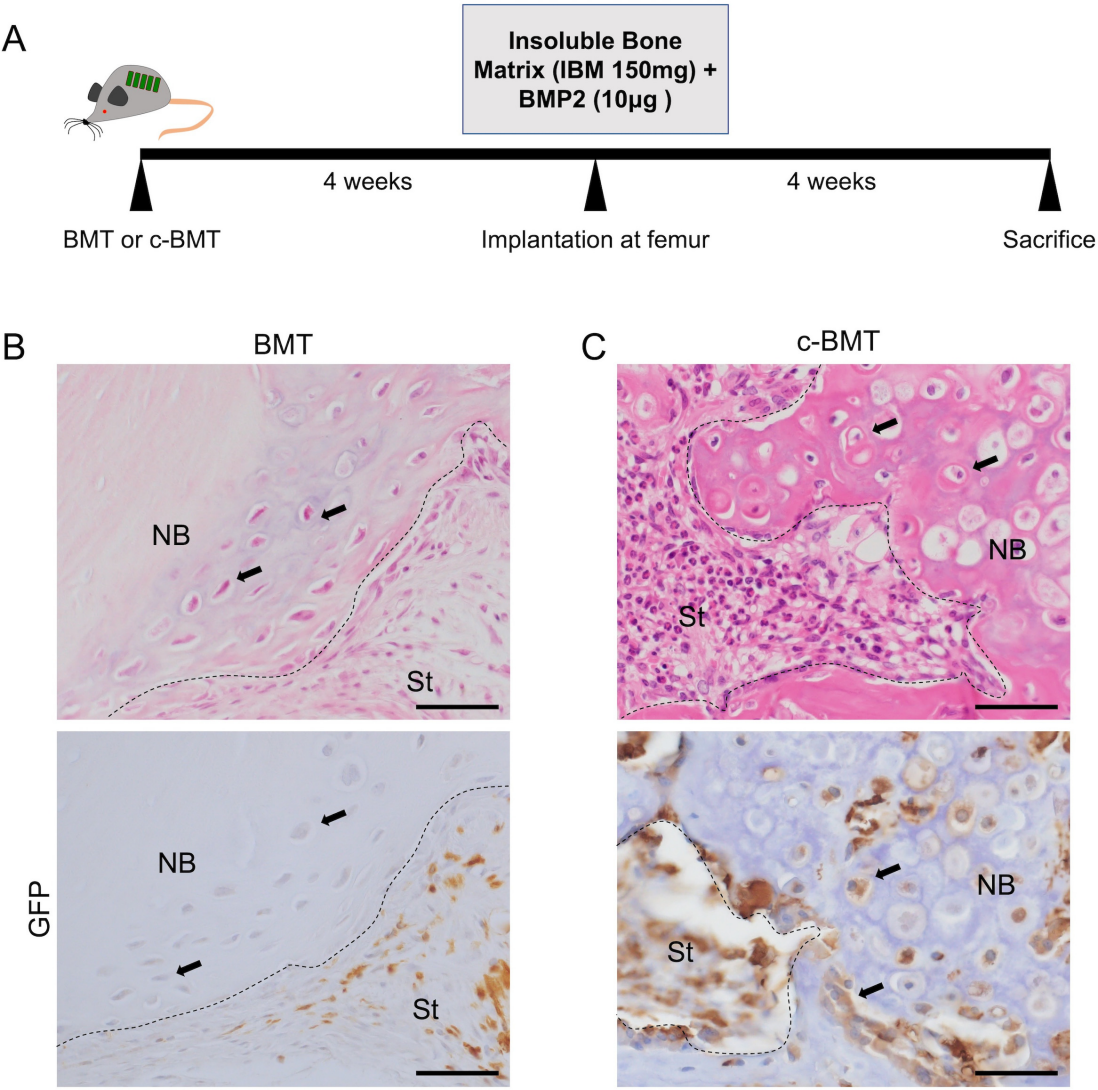
Ectopic bone formation occurred in both bone marrow transplantation (BMT) and modified BMT (c‐BMT). (*A*) Illustration of mice model with ectopic bone formation. The bone‐forming material implantation procedure was subjected to bone marrow‐transplanted mice after 4 weeks of BMT. After 4 weeks, mice were euthanized for analysis. (*B*) Representative images of H&E staining and GFP‐IHC staining on BMT mice. (*C*) Representative images of H&E staining and GFP‐IHC staining on c‐BMT mice. Scale bar = 50 μm. NB = new bone; St = stromal area. Black arrows show the chondrocytes at the new bone area.

To investigate and compare the contribution of transplanted BMSC on bone formation between modified c‐BMT and normal BMT, we implanted the bone‐forming materials: BMP2 together with insoluble bone matrix in the femur of mice after 4 weeks of BMT (Fig. 2*A*). After 4 weeks of implantation, the mice were euthanized, and histological analysis was performed. On histological examination, all BMT and c‐BMT mice showed endochondral ossification at the femur where implantation was performed (Fig. 2 *B*, *C*). We observed the cartilage formation at the periphery of the implanted materials and existing femoral bone. Connective tissue stromal area was observed and composed of spindle‐shaped fibroblasts and inflammatory cells.

### c‐BMT recruited bone marrow‐derived bone‐forming cells, but BMT did not

To check whether the cells involved in the ectopic bone formation were derived from BM, we performed GFP IHC staining on the ectopic bone‐forming area. Both BMT and c‐BMT mice showed that GFP^+^ cells were abundantly observed at the stroma area in the ectopic bone‐forming area and were rounded or spindle in shape (Fig. 2 *B*, *C*). Interestingly, while c‐BMT mice showed GFP‐positiveness on osteoblasts and chondrocytes, BMT mice failed to recruit BM‐derived bone‐forming cells. This finding suggested that the origin of bone‐forming cells—osteoblasts and chondrocytes—may differ between BMT and c‐BMT mice.

### GFP ^+^ RUNX2 ^+^, GFP ^+^Osteocalcin^+^, and GFP ^+^Sox9^+^ cells are recruited to the bone‐forming area in the c‐BMT model

**Fig. 3:**
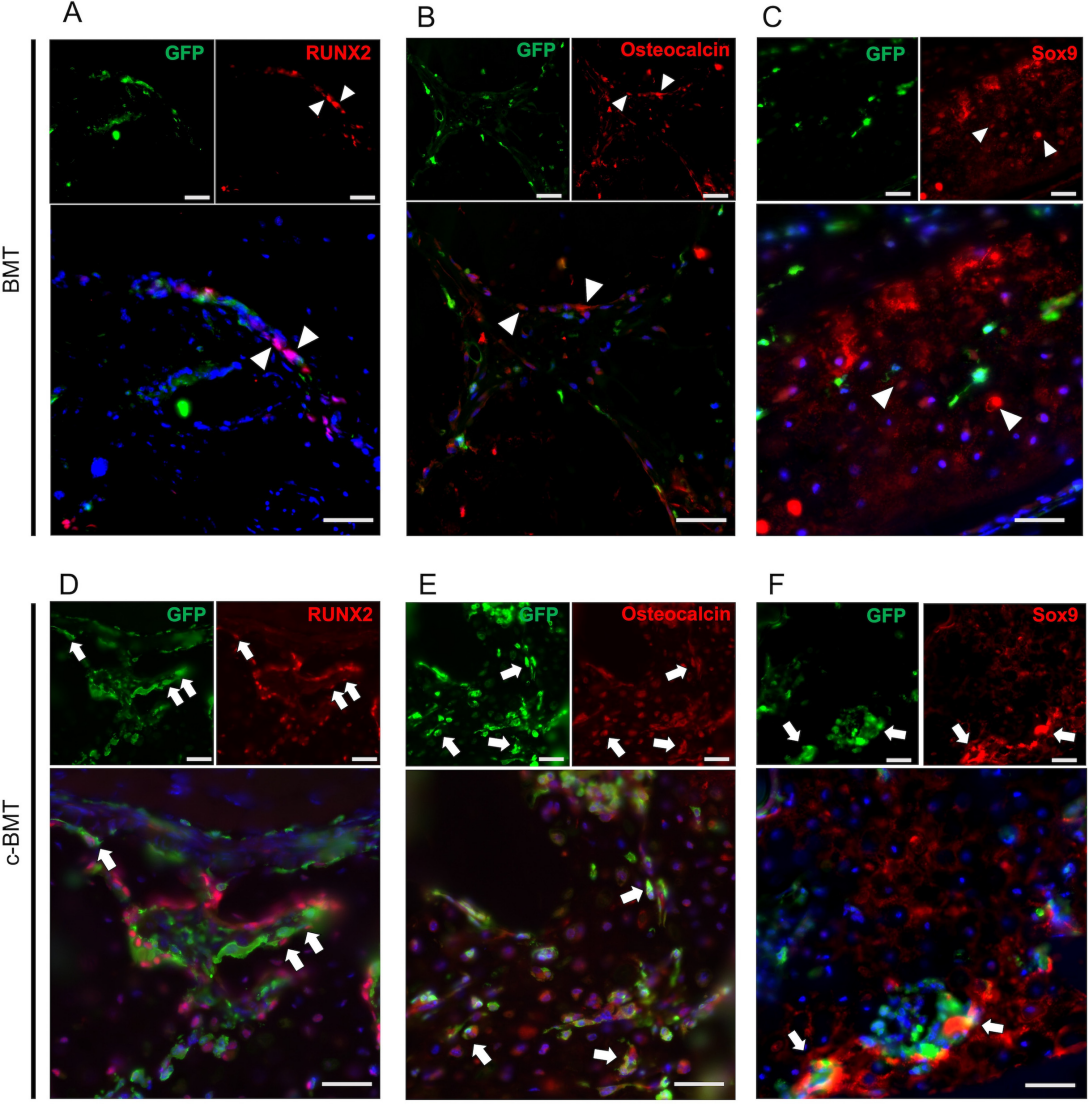
Modified bone marrow transplantation (c‐BMT) recruited bone marrow‐derived bone‐forming cells, but BMT did not. Double IHC staining on BMT mice: (*A*) RUNX2 (red) and GFP (green), (*B*) osteocalcin (red) and GFP (green), (*C*) Sox9 (red) and GFP (green). Double IHC staining on c‐BMT mice: (*D*) RUNX2 (red) and GFP (green), (*E*) osteocalcin (red) and GFP (green), (*F*) Sox9 (red) and GFP (green). Scale bar = 50 μm. White arrowheads show the single‐positive cells, and white arrows show the double‐positive cells.

Next, to confirm whether the c‐BMT model recruits the BM‐derived bone‐forming cells, we analyzed osteoblast and chondrocyte differentiation performing double‐fluorescent IHC on GFP together with RUNX2, a transcription factor of osteoblastic differentiation, or osteocalcin, a marker of mature osteoblasts, or Sox9, a marker of chondrocytes.^(^ ^18^, ^19^ ^)^ We observed colocalization of either RUNX2 and GFP or osteocalcin and GFP or Sox9 and GFP at the periphery of the new bone formation area in c‐BMT mice but not in BMT mice (Fig. 3*A–C*). These findings suggested that our modified c‐BMT technique could collect the BMSC from donor mice.

### GFP ^+^ RUNX2 ^+^ stromal cells were recruited in c‐BMT mice

**Fig. 4:**
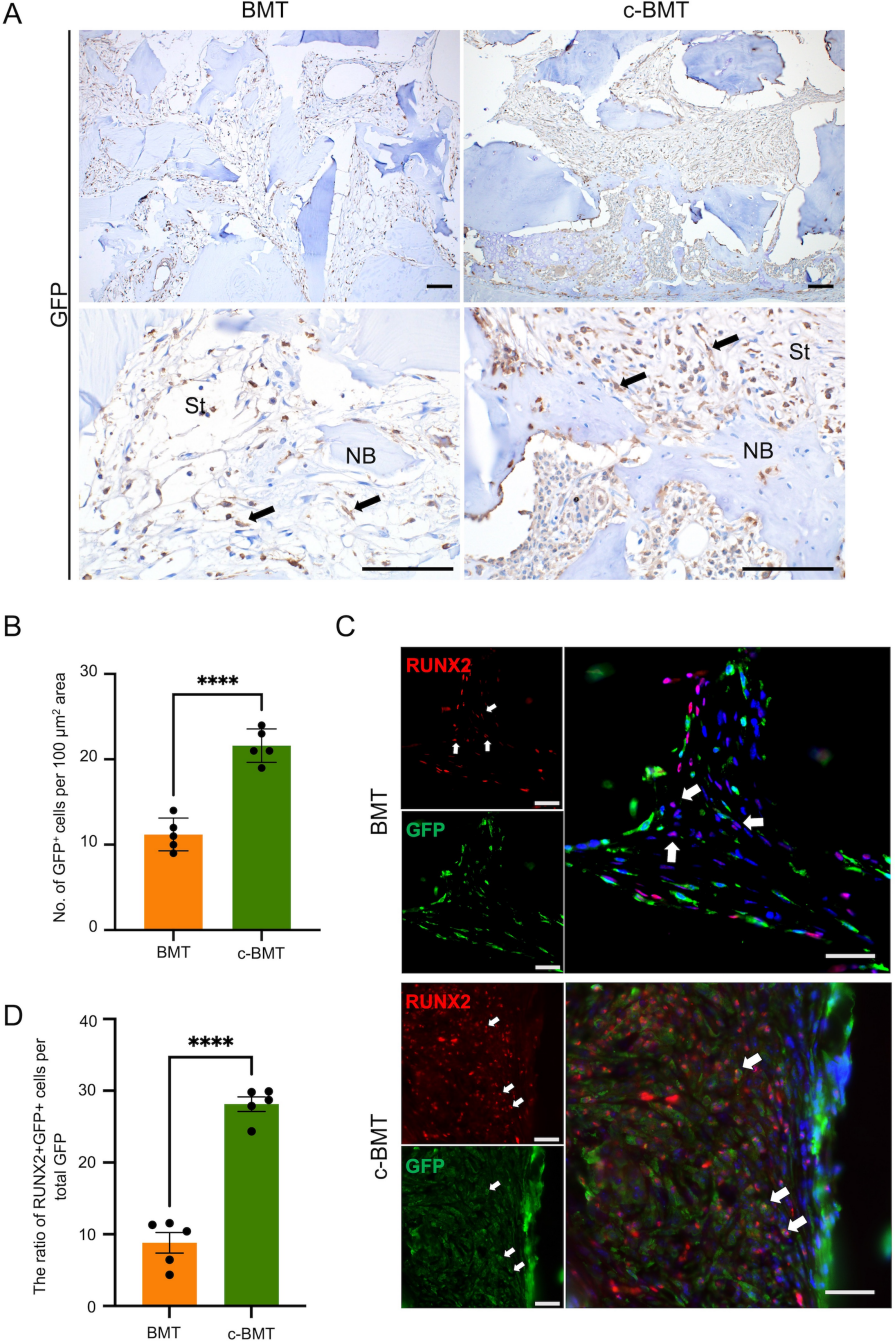
RUNX2^+^ stromal cells are recruited in modified bone marrow transplantation (c‐BMT) mice more than in BMT mice. (*A*) Representative images of GFP IHC staining. Upper panel = low magnification; lower panel = high magnification. (*B*) GFP^+^ cells in the 100 μm^2^ of stromal area. (*C*) Double IHC staining of RUNX2 (red) and GFP (green) on BMT and c‐BMT mice. (*D*) The ratio of RUNX2^+^GFP^+^ cells per total GFP. Scale bar = 50 μm. NB = new bone; St = stromal area. Black arrows show GFP^+^ cells in the stroma area. White arrows show the double‐positive cells. Statistical analysis was performed using Student's *t* test, one‐tailed; **** *p* < 0.0001. Dots in the plots represent the mean value of each mouse. Data are represented as the mean ± SD, *n* = 5.

Around the implanted materials, the connective tissue stromal area was found in both BMT and c‐BMT mice. The stromal area was composed of fibroblast‐like spindle‐shaped mesenchymal cells and immune cells. However, the recruitment of BMSC differed between normal BMT and c‐BMT mice. On GFP IHC detection, GFP^+^ cellular density is different between the two groups (Fig. 4 *A*, *B*). Then, we investigated whether these GFP^+^ mesenchymal cells have the potential to differentiate into osteoblasts by performing fluorescent IHC on GFP together with RUNX2. We observed that the BM‐derived cells produced osteoblast differentiation factor, RUNX2, in c‐BMT mice (Fig. 4*C*). On quantification, c‐BMT mice showed a significantly higher number in proportion of GFP^+^RUNX2^+^ stromal cells than normal BMT mice (Fig. 4*D*). These data indicated that c‐BMT recruited more osteoblast precursors than routine BMT.

### c‐BMT method isolated BMSC more than the routine BMT method

**Fig. 5:**
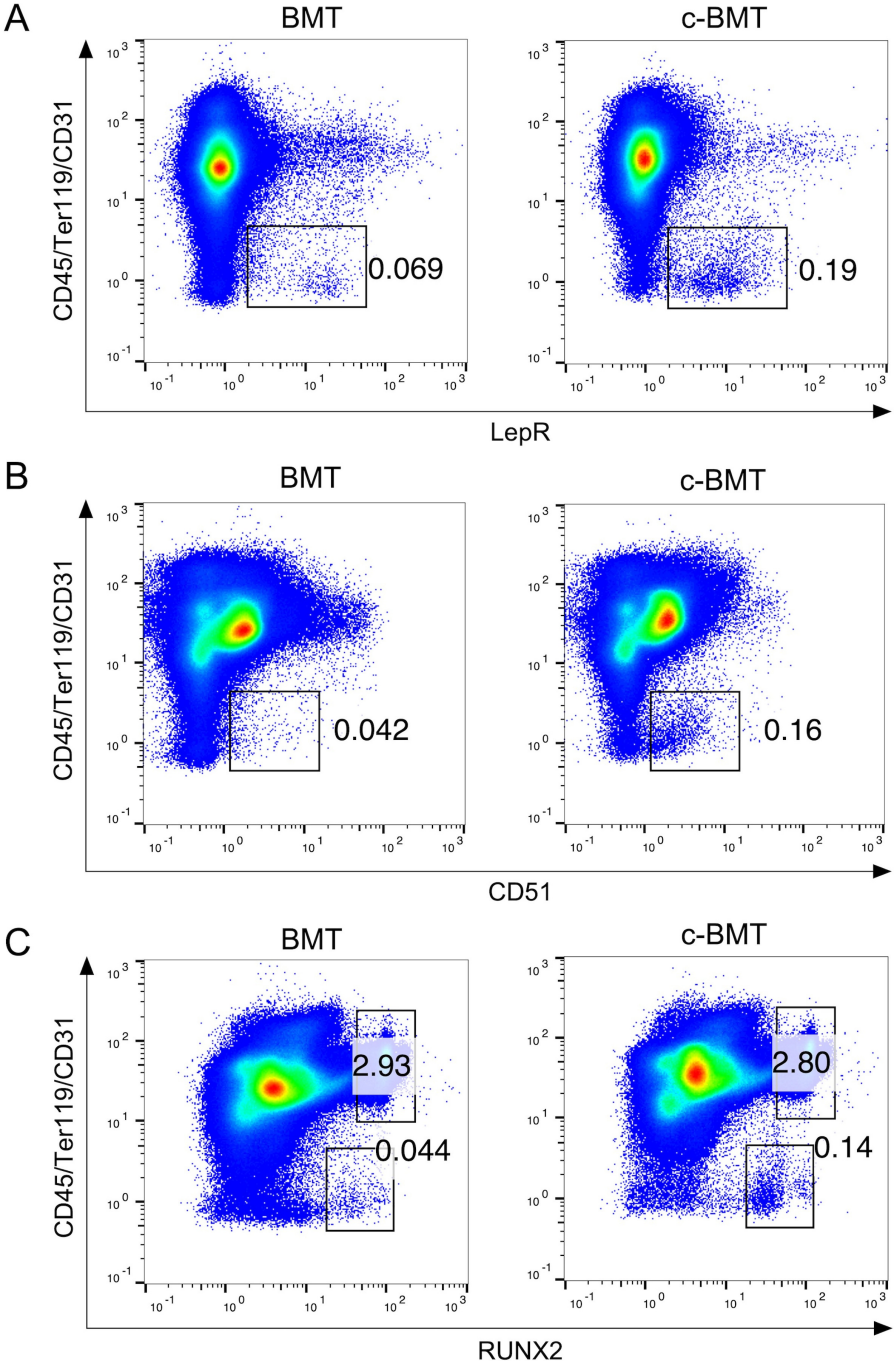
Modified bone marrow transplantation (c‐BMT) method isolated the bone marrow stromal cells more than the normal BMT method. Scatter plots from flow cytometry analysis of isolated bone marrow cells depicting CD45/Ter119/CD31‐positive and LepR^+^ (*A*), CD51^+^ (*B*), and RUNX2^+^ (*C*). Left panel: BMT; right panel: c‐BMT. Numbers indicate the percentage of LepR^+^, CD51^+^, and RUNX2^+^ cells in the total population.

Further, we performed the flow cytometry analysis to evaluate the proportion of BMSC in the collected BM cells. Then, we compared two methods. Bone marrow has been known to be the niche for the LepR^+^ stromal cells, which are responsible for the maintenance of HSCs in bone marrow.^(^ ^20^, ^21^ ^)^ These LepR^+^ stromal cells are the source of the skeletal cells and myofibroblasts.^(^ ^17^, ^22^ ^)^ In addition, CD51 is expressed in the subpopulation of bone marrow mesenchymal stem cells, and CD51^+^ BMSC facilitates the proliferation and migration where necessary.^(^ ^23^ ^)^ Therefore, we checked the expression of LepR and CD51 on isolated BM cells to evaluate whether BMSC were included. We observed that the proportion of LepR^+^ BMSC was higher in c‐BMT (0.19%) than in BMT (0.069%) (Fig. 5*A*). The purpose of c‐BMT is to facilitate the isolation of BMSC by enzymatic disaggregation of bone marrow cells, ie, to minimize the retention of the bone marrow cell aggregates on filtration. Here, we also investigated whether the enzyme treatment after the filtration step tends to isolate the bone marrow mesenchymal stem cells. Notably, enzyme treatment after the filtration step did not show a difference in the relative proportion of BMSC (Supplemental Fig. S2). These data supported our hypothesis, suggesting that collagenase and dispase treatment before filtration successfully disaggregate the BM cells and allow BMSC to pass through the filtration.

In addition, the CD51^+^ BMSC proportion was higher in c‐BMT (0.16%) than in BMT (0.042%) (Fig. 5*B*). Again, to evaluate the osteoblast differentiation ability in isolated bone marrow cells, we detected RUNX2 expression. Consistent with IHC staining, c‐BMT showed a higher proportion of RUNX2^+^ cells than the BMT method (0.14% and 0.044%, respectively) (Fig. 5*C*). These data indicated that the c‐BMT method could isolate a higher proportion of BMSC with a higher potential to produce osteoblast differentiation factors than the BMT method.

## Discussion

The bone marrow microenvironment provides the critical niches for HSCs and MSCs. The generation and mobilization of multilineage stem cells from BM is a major response to the tissue repair and regeneration of multiple organs after the injury.^(^ ^24^, ^25^, ^26^, ^27^ ^)^ The BMSC have the potential to differentiate into osteoblasts and be involved in the bone‐healing process.^(^ ^28^, ^29^ ^)^ Many studies have evaluated the differentiation of BMSC to bone tissue formation in bone marrow–transplanted mice models.^(^ ^11^, ^30^, ^31^ ^)^ However, there was a controversial finding on the differentiation of BM cells to osteoblasts at the bone‐healing site, and this finding led to raising the question of bone marrow transplantation techniques. Therefore, we speculated that the transplanted BM cell population might differ in every study. In this present study, the modified c‐BMT method sought this unsolving problem.

BMSC isolation is technically challenged. In the present study, we proposed a modified BM cell isolation method, c‐BMT. The significant modification of c‐BMT is a designation to collect the bone marrow stromal cells and mesenchymal cells from donor mice. In the bone marrow, hematopoietic stem cells expressed the adhesion molecules such as α4β1, CXCR4. They were homed together with the bone marrow mesenchymal cells and stromal cells, which produce specific ligands such as VCAM1 and CXCL12.^(^ ^32^, ^33^, ^34^, ^35^ ^)^ Consistent with this, colony aggregations of BM cells were observed when BM cells were flushed out from the bone marrow for BMT in mice. Therefore, collected BM cells were filtered to obtain the single‐cell suspension. Here, we hypothesized that this filtration procedure might limit the passage of BM cell colony (hematopoietic stem cells, bone marrow mesenchymal stem cells, and stromal cells) and result in the unsuccessful transplantation of BMSC to recipient mice. Therefore, BM cell dissociation before the filtration step would yield a greater population of BM cells and BMSC.

Previous studies showed that enzyme‐integrated bone marrow isolation provides promising results in isolating the functional BMSC.^(^ ^36^, ^37^ ^)^ Here, we also applied collagenase and dispase to dissociate the BM cells from bone marrow. Consistent with the previous studies, c‐BMT showed a notably higher proportion of LepR^+^, CD51^+^ bone marrow stromal cells than the BMT method. However, whether enzyme‐integrated isolated BMSC can survive and perform their optimal functions in the recipient has not been evaluated. To this end, we transplanted the isolated BM cells to the recipient mice, and it was observed that our isolated BM cells, including BMSC, survived in the recipient mice. In addition, RUNX2, osteocalcin, and Sox9‐expressing BM cells were observed in the ectopic bone formation area. These findings indicated that the c‐BMT method successfully collected BM stem and progenitor cells, which not only can survive but also can contribute to bone formation. Notably, enzyme treatment after filtration showed no effect on the LepR^+^ BM cell population. These data suggested that the enzymatic integration at the BM cell collection step, particularly before the filtration procedure, may be the solution for the controversial findings on the origin of the bone‐forming cells in the bone‐healing process.

Many different BMT studies were performed to evaluate the nature of BM‐derived cells and have demonstrated that BMSC differentiates into osteoblasts and chondroblasts and involves bone formation.^(^ ^30^, ^38^, ^39^, ^40^, ^41^, ^42^ ^)^ Massimo Dominici and colleagues performed the sequential procedure of bone marrow transplantation: (i) GFP transfection to isolated bone marrow cells, (ii) culture, and (iii) transplantation to recipient mice via tail vein.^(^ ^30^ ^)^ On the other hand, In‐Hong Kang and colleaguues performed single‐cell HSC transplantation and demonstrated the involvement of HSCs in osteoblast differentiation.^(^ ^38^ ^)^ Later, bone marrow cells from GFP transgenic mice were collected and transplanted to the recipient mice and evaluated bone marrow‐derived cells.^(^ ^11^, ^14^, ^43^ ^)^ Here, we reported the modified bone marrow transplantation technique, c‐BMT, which is time‐efficient. Our method is technically simple and reliable to engraft the bone marrow stem cells to the recipient mice.

HSCs transplantation is a promising approach to curing the blood disorders such as leukemia, myeloproliferative disorders, and lymphoma.^(^ ^44^ ^)^ MSC is a source of bone‐forming precursors for bone diseases and conditions, and MSC transplantation is widely used in different approaches: local or systemic.^(^ ^45^, ^46^ ^)^ It has been reported that the transplantation of isolated and in vitro proliferated MSC to osteogenesis imperfecta patients and mice showed the successful differentiation of transplanted cells to functional osteoblasts.^(^ ^8^, ^9^ ^)^ To our knowledge, current treatments of osteogenesis imperfecta aim to reduce the severity of the disease but not to cure the disease. Hence, we proposed that bone marrow transplantation might be a promising approach to replacing defective bone marrow with functional bone‐forming precursors. Our c‐BMT method might be the potential approach to cure osteogenesis imperfecta, and it needs more investigations for clinical application.

In conclusion, the present study showed the novel modified bone marrow transplantation method to improve the studies on bone marrow transplanted models to ensure the engraftment of total BM cells, including HSCs and BMSC. In addition, the present methodology is assessable and time‐efficient. Therefore, although it needs to be investigated more, this current method might be helpful for stem cell therapy where applicable in humans.

## Conflicts of Interest

The authors declare no conflicts of interest.

## Author Contributions

HK conceptualized, designed, and managed the study, performed bone marrow transplantation, immunohistochemistry, flow cytometry analysis, wrote the original draft of the manuscript, and acquired funding. MWO performed bone marrow transplantation, ectopic bone formation mice model, immunohistochemistry, flow cytometry analysis, data analysis, and wrote the original draft of the manuscript. KT performed bone marrow transplantation, ectopic bone formation mice model, data analysis, and acquired funding. IT performed bone marrow transplantation and flow cytometry analysis. YS performed immunohistochemistry and data analysis. HSE and Sho S performed data analysis. SF and Shintaro S performed experiment and acquired funding. KN edited the manuscript and acquired funding. HN and TT supervised HK and MWO. All authors have read and agreed to the published version of the manuscript.

### Peer Review

The peer review history for this article is available at https://publons.com/publon/10.1002/jbm4.10722.

## Supporting information


**Supplemental Fig. S1.** Investigation for the optimal concentration of the collagenase and dispase used in the c‐BMT method. Scatter plots from flow cytometry analysis of isolated bone marrow cells depicting CD45/Ter119/CD31‐positive and LepR^+^. Numbers indicate the percentage of LepR^+^, CD51^+^, and RUNX2^+^ cells in the total population. The blue box shows the optimal concentration for c‐BMT.
**Supplemental Fig. S2.** Enzyme treatment on BM cells after filtration did not affect LepR+ BM cell population. To investigate whether enzyme treatment differs the filtration step of BM cell isolation, we analyzed the LepR^+^ BM cell population with or without enzyme treatment after filtration. Scatter plots from flow cytometry analysis of isolated BM cells depicting CD45/Ter119/CD31‐positive and LepR^+^. (*A*) No enzyme treatment after filtration. (*B*) Enzyme treatment after filtration.
**Supplemental Table S1.** Primary Antibodies Used in Immunohistochemistry
**Supplemental Table S2.** Secondary Antibodies Used in Double‐Fluorescent Immunohistochemistry
