# Supplementary material for: Enzyme‐Cleaved Bone Marrow Transplantation Improves the Engraftment of Bone Marrow Mesenchymal Stem Cells
Source: JBMR Plus. 2023 Feb 11;7(3):e10722. doi: 10.1002/jbm4.10722 (PMC10020919; doi:10.1002/jbm4.10722)
Supplement: Supplementary file 1 — Supplemental Fig. S1. Investigation for the optimal concentration of the collagenase and dispase used in the c‐BMT method. Scatter plots from flow cytometry analysis of isolated bone marrow cells depicting CD45/Ter119/CD31‐positive and LepR+. Numbers indicate the percentage of LepR+, CD51+, and RUNX2+ cells in the total population. The blue box shows the optimal concentration for c‐BMT. Supplemental Fig. S2. Enzyme treatment on BM cells after filtration did not affect LepR+ BM cell population. To investigate whether enzyme treatment differs the filtration step of BM cell isolation, we analyzed the LepR+ BM cell population with or without enzyme treatment after filtration. Scatter plots from flow cytometry analysis of isolated BM cells depicting CD45/Ter119/CD31‐positive and LepR+. (A) No enzyme treatment after filtration. (B) Enzyme treatment after filtration. Supplemental Table S1. Primary Antibodies Used in Immunohistochemistry Supplemental Table S2. Secondary Antibodies Used in Double‐Fluorescent Immunohistochemistry [file JBM4-7-e10722-s001.docx]

**Appendix to the manuscript**

**RE: JBMR Plus MS# JBM4-08-22-0087**

**Enzyme-cleaved bone marrow transplantation improves the engraftment of bone marrow mesenchymal stem cells**

Hotaka Kawai DDS, PhD ^1,#,*^, May Wathone Oo DDS ^1,#^, Kiyofumi Takabatake DDS, PhD ^1^, Ikue Tosa DDS, PhD ^2,3^, Yamin Soe DDS ^1^, Htoo Shwe Eain DDS ^1^, Sho Sanou DDS ^1^, Shigeko Fushimi PhD ^1^, Shintaro Sukegawa DDS, PhD ^1,4^, Keisuke Nakano DDS, PhD ^1^, Takarada Takeshi PhD ^2^, and Hitoshi Nagatsuka DDS, PhD ^1^

^1^ Department of Oral Pathology and Medicine, Graduate School of Medicine, Dentistry and Pharmaceutical Sciences, Okayama University, Okayama 700-8525, Japan

^2^ Department of Regenerative Science, Graduate School of Medicine, Dentistry and Pharmaceutical Sciences, Okayama University, Okayama 700-8558, Japan

^3^ Cartilage Biology and Regenerative Medicine Laboratory, College of Dental Medicine, Columbia University Irving Medical Center; New York, NY 10032, USA.

^4^ Department of Oral and Maxillofacial Surgery, Kagawa Prefectural Central Hospital, Takamatsu 760-8557, Japan

^#^ These authors equally contributed to this manuscript.

^*^ Corresponding author:

Hotaka Kawai DDS, PhD

Department of Oral Pathology and Medicine, Graduate School of Medicine, Dentistry and Pharmaceutical Sciences, Okayama University, Okayama 700-8525, Japan

Tel.: +81-86-235-6651, Fax: +81-86-235-6654

Email: [hotaka-k@okayama-u.ac.jp](mailto:hotaka-k@okayama-u.ac.jp)


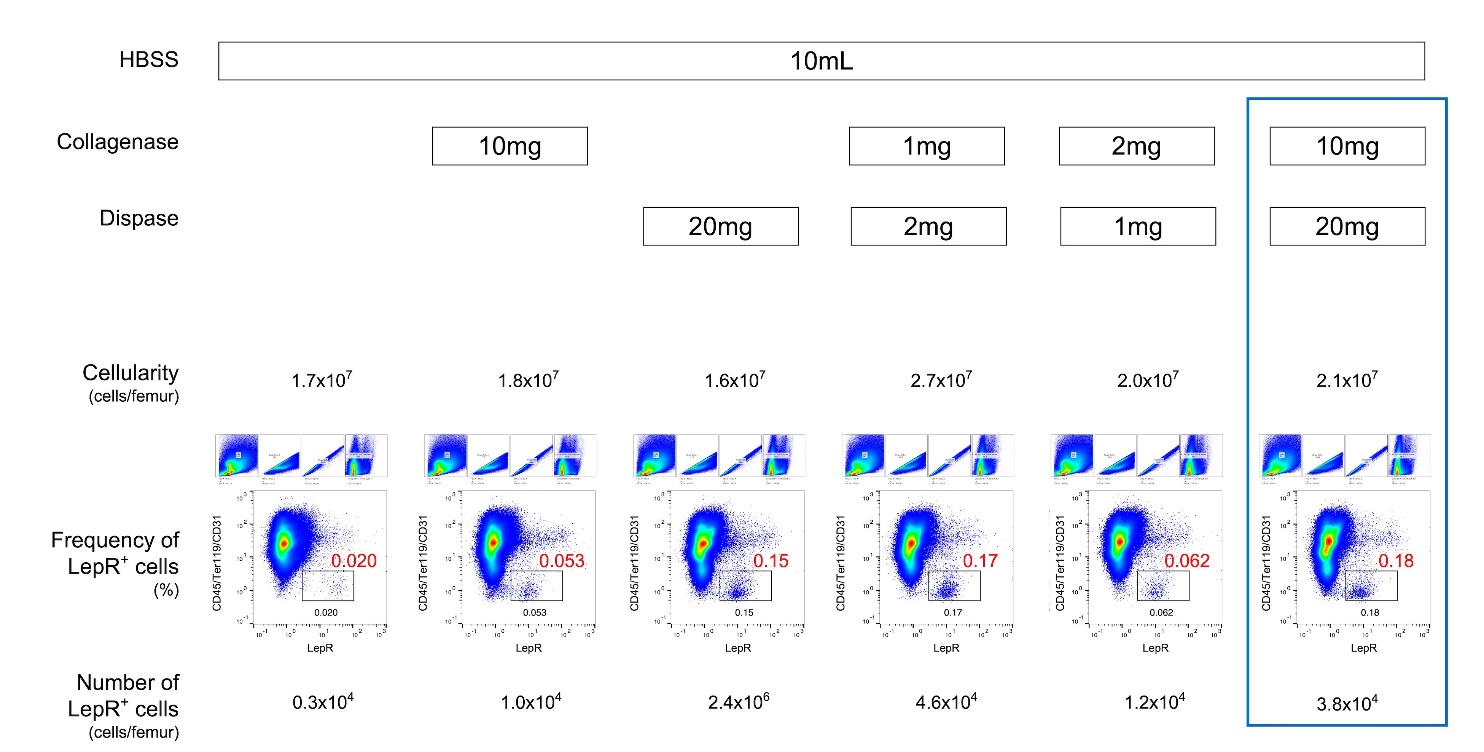


**Supplementary figure 1: Investigation for the optimal concentration of the collagenase and dispase used in the c-BMT method.** Scatter plots from flow cytometry analysis of isolated bone marrow cells depicting CD45/Ter119/CD31-positive and LepR^+^. Numbers indicate the percentage of LepR^+^, CD51^+^, and RUNX2^+^ cells in the total population. The blue box shows the optimal concentration for c-BMT.


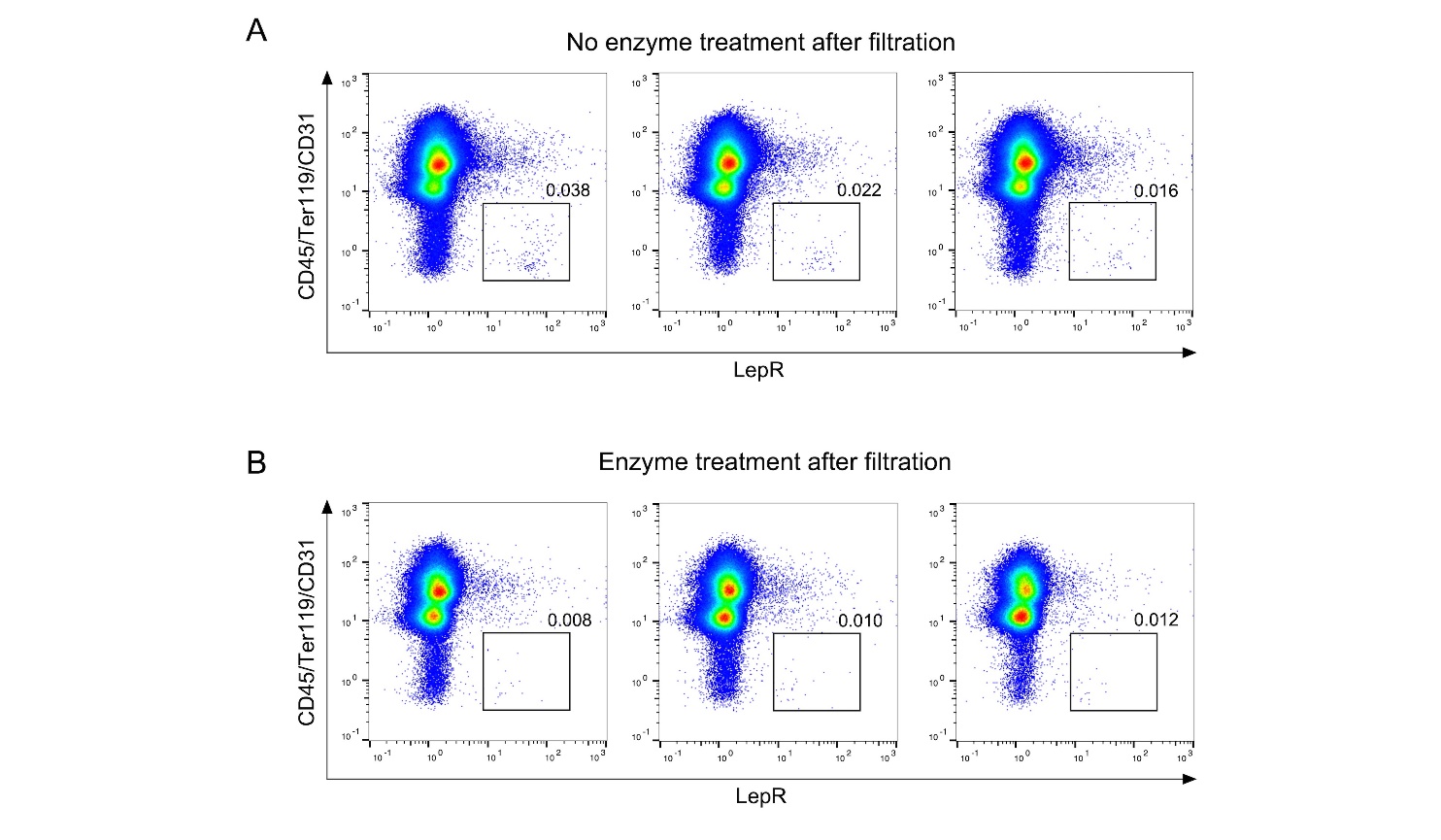


**Supplementary figure 2: Enzyme treatment on BM cells after filtration did not affect on LepR+ BM cells population.** To investigate whether enzyme treatment differs the filtration step of BM cells isolation, we analyzed the LepR^+^ BM cells population with or without enzyme treatment after filtration. Scatter plots from flow cytometry analysis of isolated BM cells depicting CD45/Ter119/CD31-positive and LepR^+^. **(A)** No enzyme treatment after filtration. **(B)** Enzyme treatment after filtration.

**Table S1: Primary antibodies used in immunohistochemistry**

| Primary antibody | Immunized animal | Antigen retrieval | Dilution | Supplier |
| --- | --- | --- | --- | --- |
| GFP | Goat | Microwave heating in 0.01 mol/L citrate buffer (pH 6.0) at 100°C for 1 min | 1:500 | Abcam  (ab6673) |
| RUNX2 | Rabbit | Microwave heating in 0.01 mol/L citrate buffer (pH 6.0) at 100°C for 1 min | 1:1000 | Abcam  (ab192259) |
| Osteocalcin | Rabbit | Microwave heating in 0.01 mol/L citrate buffer (pH 6.0) at 100°C for 1 min | 1:1000 | Takara  (005FDF) |
| Sox9 | Rabbit | Microwave heating in 0.01 mol/L citrate buffer (pH 6.0) at 100°C for 1 min | 1:500 | Abcam  (ab26414) |

**Table S2: Secondary antibodies used in double-fluorescent immunohistochemistry**

| Secondary antibody | Immunized animal | Fluorescent dye | Supplier |
| --- | --- | --- | --- |
| Anti-goat IgG | Donkey | Alexa Fluor 488 | Thermo  (Ref: A11055) |
| Anti-rabbit IgG | Donkey | Alexa Fluor 568 | Thermo  (Ref: A10042) |
